# New tomographic contribution to characterizing mesosaurid congenital scoliosis

**DOI:** 10.1371/journal.pone.0212416

**Published:** 2019-02-27

**Authors:** Tomasz Szczygielski, Dawid Dróżdż, Dawid Surmik, Agnieszka Kapuścińska, Bruce M. Rothschild

**Affiliations:** 1 Institute of Paleobiology, Polish Academy of Sciences, Warsaw, Poland; 2 Faculty of Earth Sciences, University of Silesia, Sosnowiec, Poland; 3 Carnegie Museum, Pittsburgh, Pennsylvania, United States of America; 4 Indiana University Ball Memorial Hospital, Muncie, Indiana, United States of America; Universiteit Maastricht, NETHERLANDS

## Abstract

The presence of a pathology in the vertebral column of the early Permian mesosaurid specimen ZPAL R VII/1, being one of the oldest amniotic occurrences of congenital scoliosis caused by a hemivertebra, was recently recognized. Here we provide CT data to further characterize the phenomenon. The affected hemivertebra is wedged (incarcerated) between the preceding and succeeding vertebrae. The neural canal is misshapen but continuous and the number of dorsal ribs on each side of the specimen corresponds with the number of the vertebrae, documenting its congenital (homeobox-related) derivation.

## Introduction

Hemivertebra is a type of congenital pathologies resulting either from an improper contralateral fusion of the paired vertebral anlagen (hemimetameric segmental shift) or from failed development of a vertebral anlage on one side of the body [1–4]. The Paleozoic record of congenital scoliosis remains scarce. Until recently, such malformations in the Paleozoic were known only in temnospondyl amphibians [3] and a single occurrence reported, but never fully described or figured, in an early Permian captorhinomorph [5]. Lately, Szczygielski et al. [6] described an incarcerated hemivertebra in a similarly aged mesosaurid, ZPAL R VII/1 –the first case known in that group and one of the oldest in amniotes. More recently, Turner and Sidor [7] suggested a case of a block vertebra in the sacrum of a late Permian pareiasaurian, and a possible case of a mild scoliosis in an aquatic procolophonoid reptile from the Permian-Triassic boundary, *Barasaurus besairiei* Piveteau [8] was mentioned by McMenamin [9]. The younger record of hemivertebrae is richer and includes a latest Permian or earliest Triassic brachyopid temnospondyl [10], a Triassic undetermined stereospondyl [11], Late Jurassic dryosaurid (hemimetameric segmental shift) [4] and plesiosaur [12], the Late Cretaceous hadrosaurid [13] and salamander [14], and an Oligocene nimravid cat [15]. Here, we provide new data concerning ZPAL R VII/1, including the images of the dorsal (sediment-encased) surface of the pathological region obtained using Computed Tomography.

## Material and methods

ZPAL R VII/1 is a mostly complete, ventrally exposed skeleton of a mesosaurid from the early Permian of Paraná state, Brazil, housed in the Institute of Paleobiology, Polish Academy of Sciences. The specimen was identified in the unpublished Master’s thesis of Kapuścińska [16] as *Stereosternum tumidum* Cope [17]. The specimen exhibits most of the *S*. *tumidum* diagnostic characters proposed thus far, which include: the number of dorsal vertebrae (22), the maximal dorsal rib diameter reaching approximately 50% of the vertebrae length, all haemal arches wishbone-shaped and pachyostotic, the proximal and the distal head of the humerus set at an angle of approximately 90°, the lateral centrale separate, teeth likely oval in cross-section [18–22]. No pisiforme was observed, but this may be a preservational or preparation artifact. Similarly, no interclavicle is preserved. The pelvis is damaged, making interpretation of the obturator foramen enclosure uncertain. Additionally, the specimen ZPAL R VII/1 is preserved in light beige tonstein, laminated sedimentary rock formed with volcanic ash cemented by carbonaceous matter deposited in shallow water under the wave base. This corresponds to the predominant occurrence of *Stereosternum tumidum* in shallow-water facies and its almost complete preservation, rather than to *Mesosaurus tenuidens* Gervais [23] which occurs in mass accumulation in deep-water black shales, mostly as fragmentary skeletons and isolated elements preserved as molds [18,22,24,25]. Since the exact specific identity of ZPAL R VII/1 is irrelevant for the conclusions presented here, we consider detailed taxonomical study as being beyond the scope of this contribution.

The specimen was scanned using Nikon/Metris XT H 225 ST computed tomograph housed in the Military University of Technology, Warsaw, Poland, with 1000 expositions (750 ms per exposition), using an Open Tube UltraFocus Reflection Target radiation source (approx. 3 μm spot) and a 1 mm thick copper filter. Each of the two parts of the slab (anterior and posterior) was scanned separately, and another scan focused on the pathological segment of the vertebral column was performed for greater resolution. The complete right femur and the distal head of the left femur are separate from the slab and thus were removed prior to scanning to avoid losing or damaging them. The voltage and power used were 200 kV with 7 W (anterior part of the slab) and 195 kV with 6.825 W (posterior part of the slab, close-up). The CT slices were visualized using VGStudio MAX 2.1. with 126 μm (anterior part), 111 μm (posterior part), and 70 μm (close-up) voxels. Based on the CT slices, 3D volumetric renderings were produced using programs Fiji [26], Drishti 2.4.6 [27], and MeshLab 2016 [28]. First, the contrast of CT slices was increased and the slice data were exported as raw files in Fiji. Then, a triangulated mesh was generated from the exported data and exported as a .PLY file in Drishti. The final processing and scaling of the 3D models was done in MeshLab. To generate the interactive 3D .PDF, the models were texturized and imported as .OBJ files into DAZ Studio (https://www.daz3d.com/daz_studio), and then exported as .U3D files. The .U3D files where implemented into 3D .PDF with Adobe Acrobat (https://acrobat.adobe.com). The CT scan slices and the 3D models are provided in S1–S2 Models and S1–S10 Movies.

The elements were identified and counted using both the CT data (most useful in the dorsal section of the vertebral column) and the actual specimen (most useful for the cervical section of the vertebral column).

## Results

**Fig 1:**
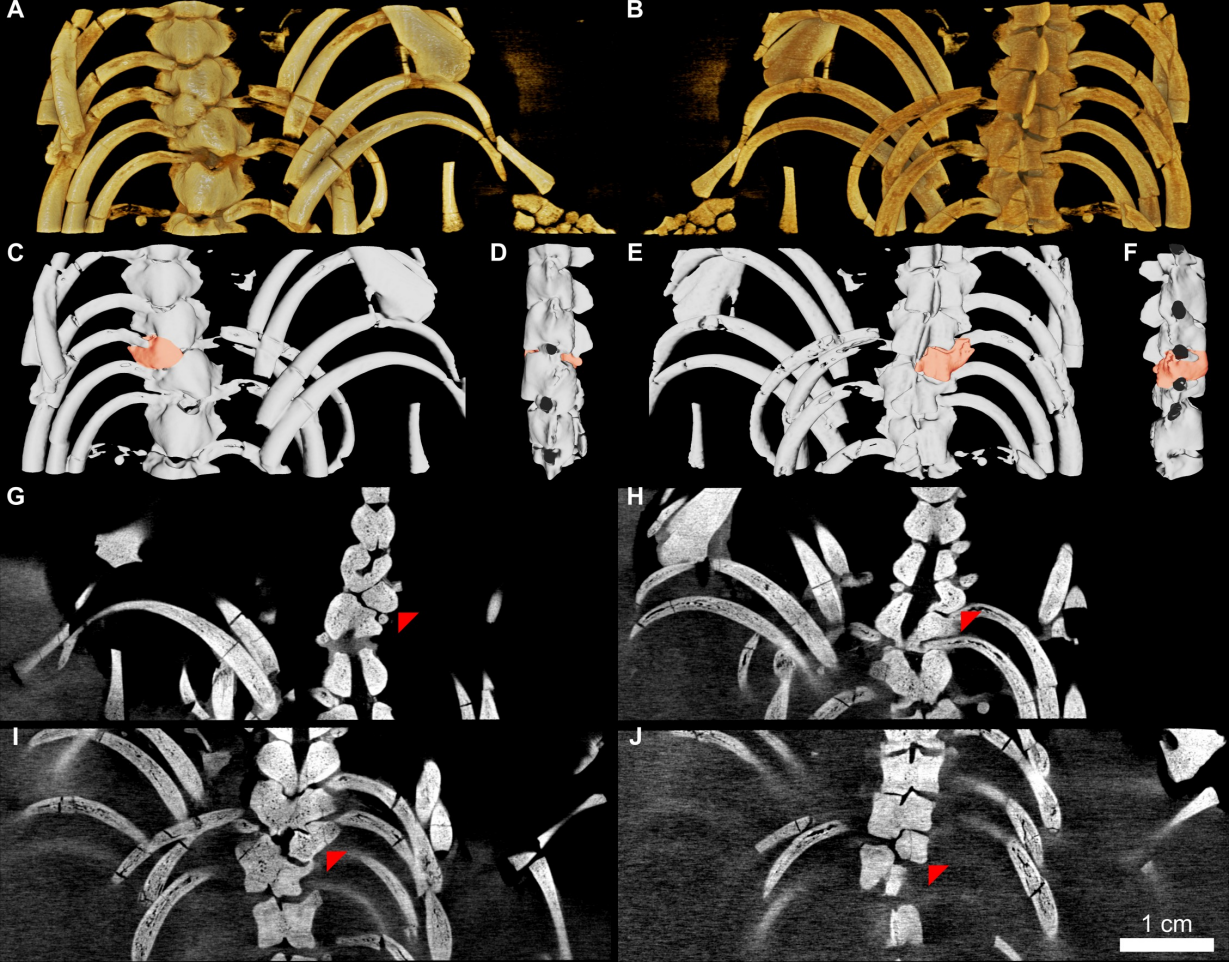
ZPAL R VII/1, patho0logical region of the vertebral column. (A-B) 3D volume render in ventral view, as physically exposed in the specimen (A) and dorsal view (B). (C-F) 3D surface render with highlighted hemivertebra in ventral view, as physically exposed in the specimen (C), lateral left (D), dorsal (E), and lateral right (F) view. (G-J) Coronal CT sections (ventral towards dorsal, 3.4 mm apart, cranial towards the top of the page, right side towards the right side of the page) showing malformed neural canal and the pathological vertebra (indicated by red arrowheads) missing its left half. Ribs and other surrounding bones removed in D and F to reveal the vertebrae.

**Fig 2:**
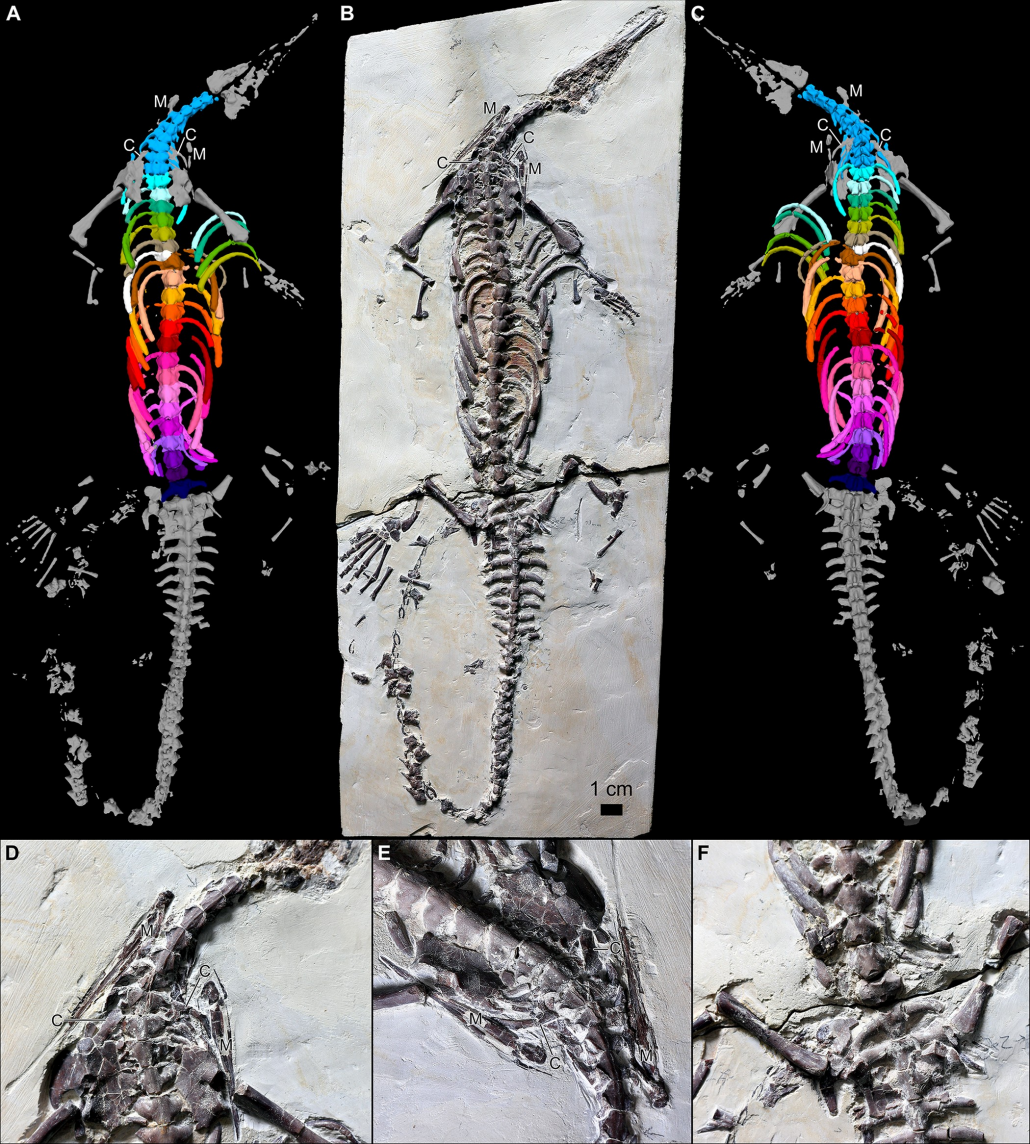
ZPAL R VII/1. (A-C) Whole specimen in ventral view (A) and as it is physically preserved (B), and in dorsal view as a volumetric render (C). (D-E) Close-ups of the cervicodorsal transition in ventral (D) and anterolateroventral (E) view. (F) Close-up of the posterior end of the dorsal region showing the break of the slab. All preserved cervical vertebrae and ribs colored using the same shade of blue, each of the dorsal vertebrae and their respective ribs colored using separate hue, the hemivertebra and the associated rib colored white. Abbreviations: C–clavicle; M–mandible. D-F not to scale.

The specimen ZPAL R VII/1, around the mid-length of its dorsum, exhibits a clear incarcerated hemivertebra [2,3,29–34], in which only half of one of the vertebrae is present. It is attached to a complete adjacent, but misshapen vertebrae [6]. As explained by Szczygielski et al. [6], the resemblance between the hemivertebra of ZPAL R VII/1 and taphonomically-disturbed mesosaurid specimens is only superficial (i.e., in both cases only a part of the affected vertebra can be observed in flattened, unilaterally exposed specimens). Closer inspection of the contact between the abnormal vertebrae of ZPAL R VII/1 reveals that the morphology does not result from post mortem dislocation or compression of the vertebrae: 1. The contact surface between them is undulating. 2. They are perfectly interlocked. 3. Their superficial features (such as fine pits) continue across the boundary. 4. Their ventral surfaces are confluent and aligned in the same plane. 5. There is no evidence of significant compaction or tectonic folding anywhere in the specimen [6]. The hemivertebra diagnosis is additionally confirmed by the CT scans of the specimen, revealing that the contralateral part of the pathological vertebrae (both the centrum and the neural arch) is completely missing (Figs 1 and 2A and 2C). Additionally, the neural canal within the conjoined vertebrae is malformed (Fig 1H). The observed morphology is unambiguously identifiable as a hemivertebra. Specimen preserves nine complete and a small fragment of a tenth cervical vertebra. At least one anteriormost cervical is likely destroyed–the skull and the anterior section of the neck are heavily damaged (Fig 2A–2D). The structures around the cervicodorsal transition are difficult to discern in the obtained images due to accumulation of more radiopaque sediment and several disarticulated and/or damaged bones at that level. Because of limited contrast and resolution, they are difficult to differentiate in the obtained slices and even in the 3D rendering. The difference between the posteriormost cervical and the anteriormost dorsal ribs is fortunately relatively prominent in mesosaurids [19], and well visualized in our data (Fig 2C) making the distinction between the neck and trunk unambiguous. The CT scans reveal 21 complete dorsal vertebrae. The hemivertebra is the 22^nd^ element of the column (Fig 2A and 2C). The nature of the contact between the hemivertebra with the neighboring vertebrae is uncertain. The exposed ventral surface shows a gently interdigitating, well-fitted, suture-like contact of the hemivertebra with the preceding centrum [6], but deeper inside, the division between these two elements is generally a straight line, showing no interdigitation (Fig 1E–1H). On the other hand, the gap between them is very narrow, so no cartilage space is evident. The obtained CT images show no obvious increase in density or sclerosis on the surfaces that would suggest movement and eburnation (polishing) of the opposing bone surfaces expected in cases of bone slippage. It is therefore possible that any movement of both bones was constrained by ligaments. It must be kept in mind, however, that the resolution and contrast of the available scans may obscure some minor features of the junction. The gap between the hemivertebra and the following vertebra is larger, more alike the normal intervertebral spaces (Fig 1E–1H), so the posterior joint might have been developed more typically.

Only 21 and 20 dorsal ribs can be identified respectively on each side–the missing pair was probably destroyed because it was associated with the second-to-last vertebra, which is located right at the break of the specimen slab (Fig 2A–2C and 2F). Impressions in the sediment around that vertebra (Fig 2F) might have originally accommodate these ribs. Likewise, the ribs of the preceding vertebra are also preserved only partially, as short rods of bone. Given that both ribs in that pair are missing, the left side of the specimen lacks a single rib compared to the right side (with the hemivertebra). It must be kept in mind, however, that the rib number alone is not the most reliable indicator for evaluation of vertebral pathologies (see below) and the morphology of the pathological vertebrae themselves is considered conclusive.

## Discussion

Identification of regions within the vertebral column of fossil amniotes is often difficult, especially in the basal taxa. The morphology and size of the ribs may change gradually, so no obvious distinctions between the cervical and dorsal region may be present and correspondence between isolated ribs and vertebrae may be difficult to ascertain [35–37]. Furthermore, significant variation (pathological or not) in the number of ribs is found within amniote populations, resulting from uni- or bilateral absence or hypoplasia of ribs [38–44], presence of supernumerary ribs [40,41,43,45–49], or their co-ossification [40,41,46,50]. Various types of scoliosis may or may not be correlated with rib aplasia, presence of additional ribs, rib malformation or asymmetry [29–32,39,41,51,52]. In some cases, ribs may fail to form an articulation with a corresponding vertebra [38]. Taphonomy [53] or even compromised recovery or preparation (e.g., accidental destruction of a bone, its removal from an otherwise complete skeleton, under-preparation causing some elements to remain unexposed within the slab) may obscure the rib count. For that reason, extreme caution must be exercised interpreting even partly disarticulated specimens. Estimation of vertebrae number based solely on the number of exposed ribs, especially in only partially prepared specimens in which some elements may be covered by other bones or sediment, may therefore be misleading [36]. Nuñez Demarco et al.’s [25] indirect (rib counting)-based claim of taphonomic origin for the published image [6] exemplifies the fallacy of such a limited approach.

Additionally, an accurate rib count for ZPAL R VII/1 cannot actually be derived (without CT assessment) from a surface image, because of incomplete preparation and pectoral girdle obscuration of the anterior rib cage. As most of the ribs are shifted to intervertebral positions and four or five left ribs are completely disarticulated, establishing correspondence with particular vertebrae cannot be performed in isolation, but requires consideration of the entire dorsal region.

Since ZPAL R VII/1 has 22 dorsal vertebrae including the hemivertebra, and the number of 22 or 23 dorsal vertebrae is considered diagnostic for *Stereosternum tumidum* [18] (specific attribution also supported for ZPAL R VII/1 by other characters), it seems evident that the hemivertebra of that specimen is an effect of an unilateral failure of a growth center rather than a supernumerary formation. According to our knowledge, no reports of a spontaneous development of supernumerary hemivertebrae are present in the literature, supporting the count of 22 dorsal vertebral segments as initial for the studied individual. Because there is only one hemivertebra present in the complete vertebral column, with all certainty the hemimetameric segmental shift was not involved.

The presence of a hemivertebra cannot simply be considered only a postural disturbance. It can directly impinge on the spinal cord with resultant weakness, paralysis and gait disturbance [54,55], but may be associated with other congenital phenomenon, in the form of arachnoid webs [56] or even diastematomyelia [57], wherein the spinal cord is split or duplicated longitudinally by a bone or cartilage spur. ZPAL R VII/1 is one of the very few fossil specimens that show a hemivertebra (not only vertebral body, but also the neural arch) in a complete vertebral column and with associated ribs. Thus, it was possible to visualize the effect of this malformation on the neural canal for the first time in a fossil. Since the individual in question attained relatively large size and advanced stage of osseous development, it seems likely that serious neurological problems did not occur in that animal, possibly due to the relieving effect of buoyancy in aquatic environment [6].

## Conclusions

The new CT data of ZPAL R VII/1 reveal a typical incarcerated hemivertebra and support the status of that specimen as the oldest case of a congenital scoliosis in an aquatic amniote (or one of the oldest, depending on the ecology and relative age of the captorhinomorph reported by Johnson [5]), as proposed by Szczygielski et al. [6].

## Supporting information

S1 Model ZPAL R VII/1. 3D model of the complete specimen.
(PDF)

S2 Model ZPAL R VII/1. 3D model of the pathological section of the vertebral column.
(PDF)

S1 Movie ZPAL R VII/1. CT slices, anterior part of the slab, caudal towards cranial (ventral surface towards the top).
(MP4)

S2 Movie ZPAL R VII/1. CT slices, anterior part of the slab, dorsal towards ventral (cranial end towards the top).
(MP4)

S3 Movie ZPAL R VII/1. CT slices, anterior part of the slab, lateral right towards lateral left (cranial end towards the top, ventral surface towards the right).
(MP4)

S4 Movie ZPAL R VII/1. CT slices, pathological section of the vertebral column, caudal towards cranial (ventral surface towards the bottom).
(MP4)

S5 Movie ZPAL R VII/1. CT slices, pathological section of the vertebral column, ventral towards dorsal (cranial end towards the top).
(MP4)

S6 Movie ZPAL R VII/1. CT slices, pathological section of the vertebral column, lateral right towards lateral left (cranial end towards the top, ventral surface towards the left).
(MP4)

S7 Movie ZPAL R VII/1. CT slices, posterior part of the slab, caudal towards cranial (ventral surface towards the top).
(MP4)

S8 Movie ZPAL R VII/1. CT slices, posterior part of the slab, dorsal towards ventral (cranial end towards the top).
(MP4)

S9 Movie ZPAL R VII/1. CT slices, posterior part of the slab, lateral left towards lateral right (cranial end towards the top, ventral surface towards the right).
(AVI)

S10 Movie ZPAL R VII/1. Spinning 3D volumetric model of the pathological section of the vertebral column.
(MP4)
