# Supplementary figures and images for: New tomographic contribution to characterizing mesosaurid congenital scoliosis
Source: PLoS One. 2019 Feb 27;14(2):e0212416. doi: 10.1371/journal.pone.0212416 (PMC6392265; doi:10.1371/journal.pone.0212416)

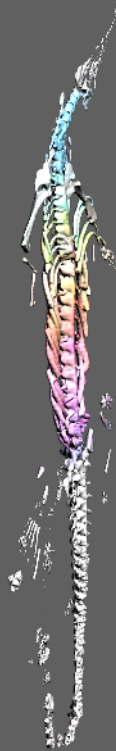

Supplement 2

Supplement: S1 Model — 3D model of the complete specimen. (PDF) [file pone.0212416.s001.pdf]

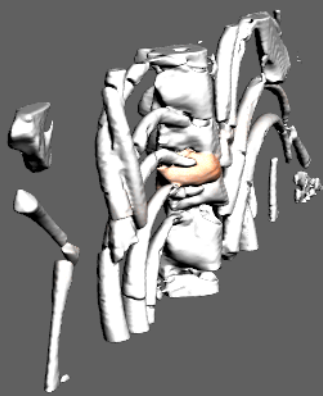

Supplement 1

Supplement: S2 Model — 3D model of the pathological section of the vertebral column. (PDF) [file pone.0212416.s002.pdf]
